# EKOS™ Jena Experience: Safety, Feasibility, and Midterm Outcomes of Percutaneous Ultrasound-Assisted Catheter-Directed Thrombolysis in Patients with Intermediate-High-Risk or High-Risk Pulmonary Embolism

**DOI:** 10.1155/2022/7135958

**Published:** 2022-02-27

**Authors:** Friederike Klein, Sven Möbius-Winkler, Laura Bäz, Rüdiger Pfeifer, Michael Fritzenwanger, Stefan Heymel, Marcus Franz, Pawel Aftanski, P. Christian Schulze, Daniel Kretzschmar

**Affiliations:** Department of Internal Medicine I, Division of Cardiology, University Hospital Jena, Jena, Germany

## Abstract

**Background:**

Percutaneous catheter-based ultrasound-assisted thrombolysis (UACDT) is recommended for patients with intermediate-high-risk or high-risk pulmonary embolism (PE) in whom systemic thrombolysis has failed or is contraindicated.

**Aim:**

To evaluate the safety and efficiency of UACDT in patients with intermediate-high-risk or high-risk PE.

**Methods:**

Between October 2017 and January 2020, we performed UACDT using the EkoSonic™ Endovascular System (EKOS™) in 51 patients (21 males, age 63 ± 18 years) with a sPESI of 1.3 ± 0.7. The EKOS™-catheter was implanted within 24 h after admission. Over 15 hours, 11.5 mg of alteplase was administered per catheter. We evaluated right ventricular stress and cardiac biomarkers before and after UACDT.

**Results:**

24 h post-UACDT, median RV/LV ratio decreased from 1.13 to 0.96 (*p* < 0.001) and the mean sPAP decreased from 47 ± 3 to 32 ± 2 mmHg + CVP (*p* < 0.0002). There were 6 major bleeding events resulting in transfusion. No stroke, myocardial infarction, right heart decompensation, or recurrent PE occurred. 31 patients (63%) were discharged without any signs of right ventricular stress. After at least 3 months, 73% of our patients did not show any signs of right ventricular dysfunction. The mean RV/LV ratio decreased to 0.75 ± 0.03 (*p* < 0.0001) in comparison with pre-UACDT, sPAP to 23  mmHg + CVP (*p* < 0.0001), and BNP to 40 pg/ml (*p* < 0.0001).

**Conclusions:**

The treatment with UACDT reduced right heart stress during the first 24 hours and midterm in patients with intermediate-high-risk or high-risk PE at an acceptable rate of severe complications.

## 1. Introduction

Pulmonary embolism (PE) is one of the most frequent cardiovascular diseases worldwide with an annual incidence of 39–115 per 100 000 population and is a major cause of cardiovascular mortality in Europe and the USA [1–4].

Usually, PE is diagnosed with high sensitivity and specificity by computed tomographic pulmonary angiography [5, 6]. Using a combination of clinical, laboratory, and imaging diagnostic parameters, the severity of the PE is classified into low risk, intermediate-low risk, intermediate-high risk, and high risk for early death [7]. The subsequent specific therapy is determined by these different risk stratifications [7].

For patients with high-risk PE or haemodynamic deterioration on anticoagulation, systemic (rescue) thrombolysis is recommended by the ESC (European Society of Cardiology) Guidelines [7] as well as by the AHA (American Heart Association) [8]. However, there are multiple contraindications, such as major trauma or surgery, active bleeding, history of haemorrhagic stroke, stroke of unknown origin or ischemic stroke during the last 6 months, bleeding diathesis, and others [7–9].

In patients with intermediate-high-risk PE, systemic thrombolysis resulted in a reduced duration of treatment on the intensive care unit as well as a reduced risk of haemodynamic decompensation and collapse but also in an increased risk of severe extracranial and intracranial bleeding [10].

There are limited data on reduced-dose systemic thrombolysis that could demonstrate similar improvements in lung perfusion and right ventricular dysfunction but significantly decrease major bleeding rates [11, 12].

Another approach using a combination of ultrasound-based fragmentation of the thrombus and catheter-directed thrombolysis, which only requires a reduced dose of the thrombolytic agent, has been developed. This localized therapy is recommended by the ESC guidelines for patients with high-risk PE and contraindications for systemic thrombolysis or intermediate-high-risk PE and haemodynamic deterioration [7].

One such device combining ultrasound and catheter-directed thrombolysis is the EkoSonic™ Endovascular System (EKOS™, Boston Scientific), which is utilized in our facility.

Data concerning ultrasound-assisted catheter-directed thrombolysis (UACDT) are promising [13]. An improvement in lung perfusion and right ventricular (RV) function and decrease in systolic pulmonary artery pressure (sPAP) and right ventricular to left ventricular (RV/LV) ratio could be shown [13–17]. There are, however, insufficient data on the midterm outcome of patients receiving UACDT.

In this publication, we present our single-center registry data with follow-up information up to six months.

## 2. Methods

Between October 2017 and January 2020, 51 patients with intermediate-high-risk or high-risk PE, in whom systemic thrombolysis was contraindicated, received UACDT using the EkoSonic™ Endovascular System (EKOS™, Boston Scientific) at our institution and were included in this retrospective registry study.

Patients either presented to the emergency department of our university hospital, were already hospitalized on various normal wards due to other diseases, or were transferred by other hospitals for advanced therapy.

After confirmation of the PE by contrast-enhanced computed tomography of the chest, right ventricular stress was evaluated using transthoracic echocardiography. Also, cardiac biomarkers (cardiac troponin I (cTNI) and brain natriuretic peptide (BNP)) were measured. At the discretion of the initially treating physician, patients received either unfractionated heparin with a target partial thromboplastin time of 60–90 seconds or weight-adjusted low-molecular-weight heparin.

If intermediate-high-risk or high-risk PE with contraindications for systemic thrombolysis was confirmed according to the ESC guidelines, patients were either admitted directly to our catheterization laboratory or accommodated on our intermediate or intensive care unit [7]. UACDT was initiated within the following 24 hours.

The EKOS™ system was implanted fluoroscopically guided in the catheterization laboratory with continuous haemodynamic and ECG (electrocardiogram) monitoring (supplement 1). Over 15 hours, 11.5 mg of alteplase was continuously administered per catheter, with a rate of 1 mg/h during the first 8 and 0.5 mg/h for the remaining 7 hours. After 15 hours of rtPA (recombinant tissue plasminogen activator) infusion and EKOS™ ultrasound, the therapy was stopped, the EKOS™ catheter was removed, and the puncture site was compressed using a femoral compression system.

Post-UACDT, patients received unfractionated heparin for at least 48 hours and were then switched to vitamin K antagonists, direct anticoagulants, or low-molecular-weight heparin. Finally, patients were transferred to a normal ward.

Right ventricular stress was evaluated using transthoracic echocardiography at baseline, within the first 24 hours after UACDT and again before discharge. In addition, cardiac biomarkers (cTNI and BNP) were followed up 24 hours after EKOS™ treatment and before discharge.

For midterm evaluation, patients were followed up for at least 3 months in our outpatient department. A physician interviewed all patients, and a blood sample was drawn in order to measure cardiac biomarkers (cTNI and BNP). Patients also received a 6-minute walking test with a blood gas analysis. Right ventricular stress was evaluated using transthoracic echocardiography.

### 2.1. Statistical Analysis

Statistical analysis was performed with the IBM SPSS Statistics version 25 for Windows. Discrete variables are presented as numbers with percentages and continuous data are presented as mean ± standard deviation or in case of a skewed distribution as median values with ranges. The comparison of within-group data was performed with the 2-sided paired *t*-test for variables, which were normally distributed. Otherwise, the Mann–Whitney *U*-test was applied. A *p* value <0.05 was considered statistically relevant.

## 3. Results

### 3.1. Study Population

51 patients received UACDT. 30 of those were women (59%), the mean age was 63 ± 18 years, and the mean BMI (body mass index) was 31.1 ± 7.1 kg/m^2^.

A deep vein thrombosis was present in 34 patients (67%), and the left leg was affected more frequently (65%). 12% of the patients had previously been diagnosed with PE and 18% with deep vein thrombosis. In 47% of our patients, a trigger for venous thromboembolism, such as immobilization, hormonal contraception, hereditary thrombophilia, or cancer was determined.

Baseline characteristics are summarized in Table 1.

### 3.2. Initial Presentation

Clinical parameters at admission are listed in Table 2.

In 88% both central pulmonary arteries were affected. The simplified PESI (pulmonary embolism severity index) was calculated at 1.3 ± 0.7 points, indicating a 30-day mortality risk of at least 10%. The initial cTNI median value was 100.0 pg/ml (range 1.3 to 1291.3 pg/ml; standard value <15.6 pg/ml). The mean initial BNP was 462.6 ± 63.9 pg/ml (standard value <100 pg/ml). The initial RV/LV ratio measured by thoracic echocardiography was 1.15 ± 0.03. The mean sPAP by echocardiography was estimated at 47 ± 3 mmHg + CVP (central venous pressure). The mean pulmonary pressure detected by catheter was 38 ± 11 mmHg.

Before UACDT, 19 patients (37%) received low-molecular-weight heparin and 32 patients received unfractionated heparin, respectively. Post-UACDT, all patients received unfractionated heparin for at least 48 hours according to our standard protocol.

### 3.3. Ultrasound-Assisted Catheter-Directed Thrombolysis (UACDT)

A cumulative dose of administered rtPA 11.5 mg (range 6 to 23 mg) was administered. 8 patients (16%) received bilateral UACDT: seven of those with a rtPA dose of 23 mg and one with 10 mg (due to premature termination of UACDT after 5 hours because of bleeding).

The median duration of UACDT was 15 hours (range 5 to 15 hours). The choice of catheter length and placement was left to the discretion of the physician. In 16%, the EKOS™ catheters were implanted bipulmonary. Nine times a 6 cm and seven times a 12 cm long catheter was used. For the remaining patients, one catheter was sufficient (median length 12 cm, range 6 to 12 cm).

### 3.4. Post-UACDT Evaluation after 24 Hours and Consecutive Treatment

Posttreatment echocardiography took place within 24 hours after the end of the UACDT procedure. The RV/LV ratio decreased significantly from 1.13 (range 0.9 to 1.4) to 0.96 (range 0.6 to 1.4, *p* < 0.001), and sPAP decreased significantly from 47 ± 3 to 32 ± 2 mmHg + CVP (*p* < 0.0002). BNP decreased from 463 ± 64 to 407 ± 83 pg/ml, although not significantly. cTNI remained stable during the first 24 hours after UACDT.

**Figure 1:**
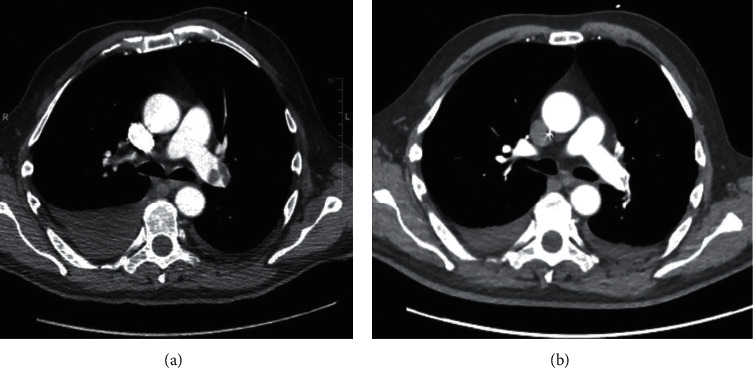
Contrast-enhanced computed tomography of the chest demonstrating a bilateral central pulmonary embolism pre-UACDT (a) and post-UACDT (b).

**Figure 2:**
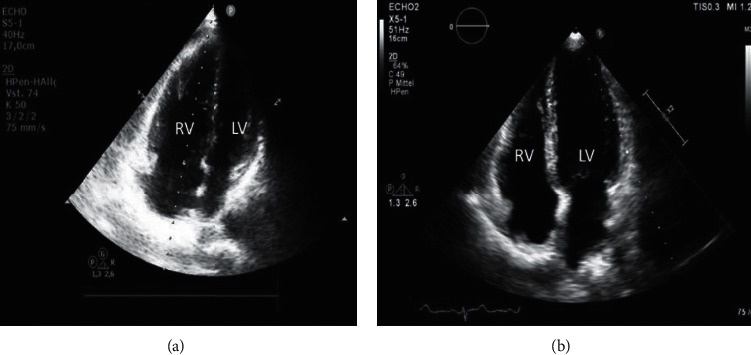
Transthoracic echocardiography, apical four-chamber view, dilated right ventricle RV pre-UACDT (a) and normal size post-UACDT (b) compared to normal-sized left ventricle (LV).

The first patients in our registry received also a contrast-enhanced computed tomography of the chest for follow-up. Examples for thrombus load pre- and post-UACDT in computer tomography and right ventricular dilatation in transthoracic echocardiography are presented in Figures 1 and 2, respectively. Due to radiation exposure, we decided to defer contrast-enhanced computed tomography scans after a few cases.

Patients stayed on the intensive or intermediate care unit for 4 ± 6 days before being transferred to a normal ward. The mean length of hospital stay was 11 ± 6 days, during which a thorough examination concerning underlying trigger factors and coexisting diseases took place.

Anticoagulation was switched after 48 hours of unfractionated heparin to edoxaban in 23 patients (45%), 9 received rivaroxaban, 8 received apixaban, 7 received low-molecular-weight heparin, and one received a vitamin k antagonist.

### 3.5. Complications

**Figure 3:**
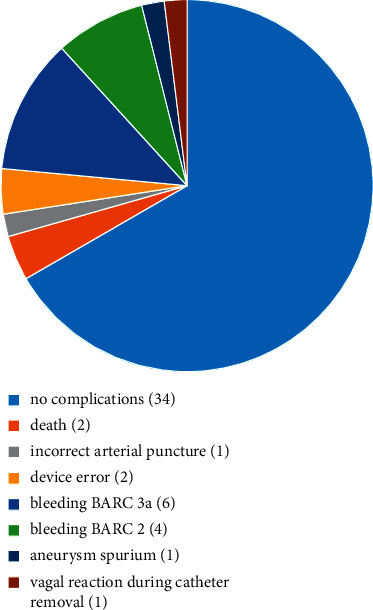
Complications. 34 of 51 patients did not suffer any complications. 2 patients died during hospitalization. There were 6 major and 4 minor bleedings. BARC = Bleeding Academic Research Consortium.

The complications are summarized in Figure 3. During hospitalization, there were two deaths, both due to pneumonia-associated sepsis. There was one accidental arterial puncture which could be treated by manual compression. Twice, a device error occurred and the ultrasound had to be terminated (after 5 and 6 hours, respectively); however, local thrombolysis could be continued.

Bleeding events were classified using the BARC (Bleeding Academic Research Consortium) scale [18].

There were 6 major bleeding events that resulted in transfusion (BARC 3a). Another 4 patients developed minor bleeding events BARC type 2.

Of the 10 bleeding events, the puncture site was afflicted seven times. Additionally, once the opposite groin area was involved. There also was one retroperitoneal bleeding and one patient developed hematochezia.

All bleeding events could be treated conservatively. No surgical intervention was necessary.

One patient showed an aneurysm spurium which was successfully treated with ultrasound assisted manual compression. One patient showed a short vagal reaction during catheter removal.

49% of our patients developed an infarction pneumonia.

There was no stroke, myocardial infarction, right heart decompensation, or recurrent PE.

### 3.6. Discharge

31 patients (60%) could be discharged without any signs of right ventricular stress determined by transthoracic echocardiography, whereas 18 still showed signs of right ventricular dysfunction. One patient with preexisting chronically obstructive pulmonary disease had to be discharged with continuous long-term oxygen therapy due to partial respiratory failure. 3 patients were diagnosed with cancer of various origins (renal cell carcinoma, breast cancer, and oral cancer, respectively) during the hospital stay.

### 3.7. Follow-Up

The majority of our patients (*n* = 38, 78%) underwent postinterventional evaluation in our outpatient clinic after three to six months.

All patients were on anticoagulation therapy with either low-molecular-weight heparin, vitamin K antagonists, or direct oral anticoagulants. No patient reported a major bleeding event.

**Figure 4:**
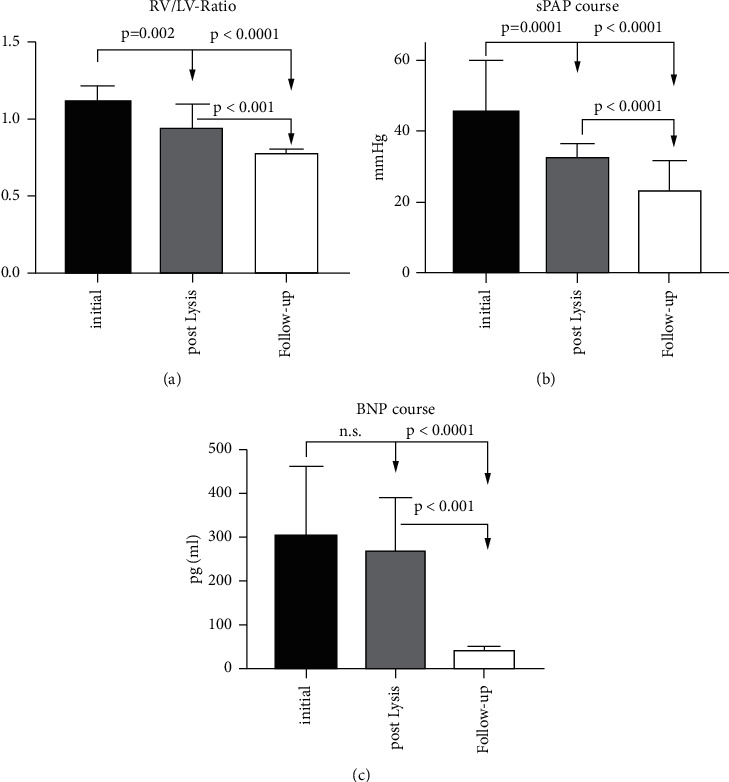
Changes of RV/LV ratio (a), sPAP (b), and BNP (c) during the observation period. RV/LV = left ventricle to right ventricle; sPAP = systolic pulmonary artery pressure; BNP = brain natriuretic peptide.

28 (73%) did not show any signs of right ventricular dysfunction by transthoracic echocardiography. In comparison to pre-UACDT, the RV/LV ratio decreased significantly from 1.5 ± 0.03 to 0.75 ± 0.03 (*p* < 0.0001), sPAP from 45 mmHg (range 20 to 80 mmHg) + CVP to 23 mmHg (range 6 to 61 mmHg, *p* < 0.0001) + CVP, and BNP from 304 pg/ml (range <10 to 2590 pg/ml) to 40.0 pg/ml (range <10 to 225 pg/ml, *p* < 0.0001, Figure 4).

The mean walking distance was 432 ± 43.5 m. Blood gas analysis was normal in 93% of our patients. Only twice there was a mild hypoxemia not requiring continuous long-term oxygen therapy. In both cases, no pulmonary hypertension and no signs of right ventricular stress were found so that other causes have to be considered. No hypercapnia was diagnosed.

Overall, 36 (73%) of our patients showed no signs of right ventricular stress after UACDT either at discharge or during follow-up.

One patient of our collective developed chronic thromboembolic pulmonary hypertension (CTEPH) and is currently treated with riociguat without need for continuous long-term oxygen therapy. Four other patients are currently evaluated for CTEPH.

## 4. Discussion

In this study, we present our single-center, registry data from a real-world population of subjects with intermediate-high-risk or high-risk PE treated at our institution. We demonstrate that UACDT treatment reduces right ventricular stress estimated by transthoracic echocardiography determining RV/LV ratio and sPAP during not only the first 24 hours but also midterm follow-up.

Regarding the short-term reduction of right heart stress, our outcomes are similar to those shown previously by the ULTIMA (Ultrasound Accelerated Thrombolysis of Pulmonary Embolism) and SEATTLE-2 (A Prospective, Single-Arm, Multi-center Trial of EkoSonic™ Endovascular System and Activase for Treatment of Acute Pulmonary Embolism) trials as well as by another single-center experience of 141 patients treated with UACDT from Kaymaz et al. [14, 15, 19].

In the ULTIMA and SEATTLE-2 trials, the mean decrease in RV/LV ratio from pre-UACDT to 24 hours post-UACDT was 0.3 and 0.42, respectively [14, 15]. Kaymaz et al. showed a mean RV/LV ratio reduction of 0.27 [19]. In our study population, the median decrease in RV/LV ratio from baseline to 24 hours post-UACDT was 0.24. Similarly, the mean reduction of sPAP from pre- to post-UACDT was 9.8 mmHg in the ULTIMA trial and 14.4 mmHg in the SEATTLE-2 study, respectively. In our collective, the decrease in sPAP from baseline to post-UACDT was 14.3 mmHg, in the Kaymaz collective 17.8 mmHg, respectively [19].

Concerning midterm outcomes, our results again are comparable to those of the ULTIMA trial. In the SEATTLE-2 trial, follow-up data were not described. In the ULTIMA study population, the mean decrease in RV/LV ratio from baseline to follow-up was 0.35. The mean reduction of sPAP from pre-UACDT to follow-up was 12.3 mmHg. In our study collective, the mean decrease in RV/LV ratio from baseline to follow-up was 0.41, and the median reduction of sPAP from pre-UACDT to follow-up was 22 mmHg.

In both collectives, there were no recurrent venous thromboembolism events during the follow-up period. All patients in both collectives were on anticoagulant therapy at the time of the follow-up visit. No bleeding events occurred during the time from discharge to follow-up visit.

Interestingly, we could show similar significant reduction in right heart stress using a lower rtPA dose than three trials mentioned above (11.5 mg compared to 24 (ULTIMA), 21 (SEATTLE-2), and 36 (Kaymaz et al.)) mg, respectively). This is mainly due to the fact that, in the Jena population, a relatively low rate of bilateral catheter use (16% compared to 87% in ULTIMA and 86% in SEATTLE-2 collective, respectively) was necessary. In the Kaymaz collective, the rate of bilateral catheterization was about 94% for patients with high-risk pulmonary embolism and 78% for those with intermediate-high-risk pulmonary embolism, respectively [19].

Two patients in our collective died due to pneumonia-associated sepsis. No procedure-associated deaths occurred. Both patients of our collective that died during hospitalization also showed severe comorbidities. One was morbidly obese (BMI 53.1 kg/m^2^) and the other had active lung cancer. The mortality rate in our collective is slightly higher than that in the SEATTLE-2 collective (3.9% vs. 2.7%) but lower than in the Kaymaz collective (5.7%). In the ULTIMA collective, no deaths were reported.

In our study population, we observed no fatal or intracranial bleedings. Major bleedings (BARC classification 3a) occurred in 11.7%, which is slightly more often than in the SEATTLE-2 trial (10%). However, no major bleeding was reported in the ULTIMA trial. Kaymaz et al. reported a major bleeding rate of 7.8% with 3 patients dying due to major bleeding [19].

4 of the 6 major bleeding incidents resulting in transfusion in our collective occurred in patients older than 80 years, a group that was excluded from the ULTIMA trial [14]. Also, our collective was slightly older than the SEATTLE-2 population (59 compared to 63 years), which could be causal for this observation [15].

Especially, the ULTIMA and the SEATTLE-2 collectives were highly selected groups, which excluded patients with a high bleeding risk, previous surgery within 7 or 10 days (SEATTLE-2 and ULTIMA, respectively), or age above 80 years (ULTIMA), for example. In our real-world collective, prior surgery or a certain age were no exclusion criteria.

In a meta-analysis of 15 trials concerning treatment of pulmonary embolism using UACDT, the major bleeding rate lays at 5.5% [13]. Another review of 28 studies showed a major bleeding rate of 5.4% [20]. The patients in both meta-analyses were slightly younger than our collective, as well. The pooled mortality rates were about equal to the studies mentioned above and our own with 3.2% and 2.9%, respectively [13, 20].

The criteria for minor and major bleeding differ between each trial. In the SEATTLE-2 study, bleeding events were defined by the GUSTO (Global Utilization of Streptokinase and Tissue Plasminogen Activator for Occluded Coronary Arteries) classification [21]. The ULTIMA trial described major bleeding as “overt bleeding associated with a fall in the hemoglobin level of at least 2.0 g/dL or with transfusion of ≥2 U of red blood cells or involvement of a critical site (intracranial, intraspinal, intraocular, retroperitoneal, intra-articular or pericardial, or intramuscular with compartment syndrome).” Bleedings that did not fulfill these criteria were classified as minor [14]. In our study, we used the BARC classification, a strongly hierarchical scale which was developed for the use in cardiovascular trials in a broad clinical context [18]. All classification systems vary at least slightly from each other so that a direct comparison between the different bleeding results in each study is difficult. Currently, there is no bleeding scale validated for patients with PE who received a systemic or catheter-directed thrombolysis.

There is also no score to predict the bleeding risk and location for patients with pulmonary embolism [8].

However, bleedings remain a major problem when performing a (rescue) thrombolysis. Also, the question whether to apply systemic thrombolysis, catheter-directed thrombolysis, or ultrasound-assisted catheter-directed thrombolysis is unresolved.

In the PEITHO trial, systemic thrombolysis with tenecteplase and heparin (compared to placebo and heparin) proved to be efficient in preventing haemodynamic decompensation in patients with intermediate-high-risk PE, but at the price of an increased risk of major bleedings and stroke. Candidates for a “safe” systemic thrombolysis could not be identified [10].

There are limited data on reduced-dose systemic thrombolysis, usually about half of the full dose infused within 2 hours, that could demonstrate similar improvements in lung perfusion and right ventricular dysfunction but at significantly lower major bleeding rates [11, 12, 22].

As stated above, the contraindications for systemic thrombolysis are numerous [7, 8]. For those patients, UACDT can be an option. However, here also, the optimal fibrinolytic dose and duration of treatment is currently under review.

In the ULTIMA and SEATTLE-2 trials, as well as in our collective, different lysis regimens were used. In the ULTIMA trial, rtPA was administered per catheter at a rate of 1 mg/h for the first 5 hours and after that 0.5 mg/h for the following 10 hours, resulting in a treatment duration of 15 hours [14]. In the SEATTLE-2 study, for patients with one catheter, rtPA was administered at a rate of 1 mg/h for 24 hours. Patients with two devices received 1 mg/h for 12 hours per catheter [15]. In our study population, the infusion rate per catheter was 1 mg rtPA per hour for the first 8 hours; during the following 7 hours, patients received 0.5 mg rtPA per hour.

The OPTALYSE trial compared 4 regimens, using 4 to 12 mg rtPA per catheter within 2 to 6 hours in 101 patients with intermediate-risk PE [16]. In 86% of the cases, 2 catheters were necessary. Bleeding events were classified using yet another bleeding scale by the ISTH (International Society on Thrombosis and Haemostasis [23]. Only in the first arm (4 mg rtPA per catheter over 2 hours), no major bleeding occurred. In the fourth arm (6 mg rtPA per catheter over 12 hours), there were 2 intracranial haemorrhages which led to the premature termination of randomization to this arm. The major bleeding rate in all groups put together was 4%. In all groups, the RV/LV ratio decreased significantly.

So, lower rtPA doses infused over shorter time periods also provide an improvement in right heart stress and result in low major bleeding rates. However, again larger studies are needed.

Many of the bleeding events in the discussed trials as well as in our own study occurred at the puncture side. To avoid this complication, ultrasound-assisted puncture could be useful, as was shown also for the puncture of the femoral artery in patients with acute limb ischemia and is now recommended by the European Society for Vascular Surgery (ESVS) [24, 25]. It is also important that UACDT should be performed in established centers with high expertise.

Another item that has to be investigated is the difference between catheter-directed thrombolysis with or without ultrasound assistance. Besides UACDT, there are a number of other catheter-directed treatment options, such as fragmentation, aspiration (e.g., AngioVac™, AngioDynamics or FlowTriever™, Inari or Indigo™ Aspiration System, Penumbra, Inc.), and rheolytic therapy (e.g., AngioJet™, Boston Scientific) although especially the latter should be used with caution due to high complications rates [26].

In theory, the greatest advantage of UACDT over catheter-directed thrombolysis without ultrasound assistance is a more effective penetration of the thrombolytic agent and therefore a shortened treatment time [8].

On this subject, there are only limited data to date. The PERFECT trial, for example, found no advantage for either treatment over the other [16]. Another trial that was investigating this aspect is the SUNSET sPE study (Standard vs. Ultrasound-assisted Catheter Thrombolysis for Submassive Pulmonary Embolism). First results were published as abstract in November 2020 [27]. Again, there were no significant differences in the reduction of RV/LV ratio. However, only in the UACDT group, two major bleeding events occurred.

Numerous open questions concerning the catheter-directed treatment of PE in general and the UACDT in particular remain. However, also mid- and long-term outcomes, concerning hard clinical endpoints such as mortality and morbidity (e.g., CTEPH) need to be evaluated [8].

## 5. Limitations

In our study, we provide real-world data concerning the treatment of high-risk and intermediate-high-risk PE using UACDT. Not only short-term outcomes were of interest but also midterm outcomes. Limiting is the small number of patients, the lack of a control group without UACDT, and the loss to follow-up of about a quarter of our study population. The remaining collective showed satisfactory outcomes after up to six months, as described above.

However, large, randomized, controlled studies and more real-world data are needed. Relevant items are, for example, the optimal duration and dosage of the thrombolysis, the necessity to combine local thrombolysis with ultrasound, and the benefit of a combination of multiple catheter-directed techniques.

## 6. Conclusion

The treatment with UACDT reduced right heart stress during not only the first 24 hours but also midterm follow-up in patients with intermediate-high-risk or high-risk PE. The rate of severe bleeding complications was acceptable. Further studies concerning the necessary dosage of the thrombolytic agent and treatment duration as well as mid- and long-term outcomes are needed.

## Figures and Tables

**Table 1:** Baseline characteristics.

	*N* = 51 (%)
Age	63 ± 18 years
Women	30 (59%)
Men	21 (41%)
BMI (kg/m^2^)	31.1 ± 7.1 kg/m^2^
Trigger	24 (47%)
Immobilization	14 (27%)
Cancer, active	9 (18%)
History of cancer	3 (6%)
Nicotine, active	4 (8%)
History of nicotine abuse	3 (6%)
Hormonal contraception	7/30 (23%)
Hereditary thrombophilia	2 (4%)
Mutation (heterozygous)	1 × Factor V Leiden
	1 × prothrombin
Deep vein thrombosis	34 (67%)
Left leg affected	22 (65%)
Recurrent venous thromboembolism	15 (29%)
Recurrent pulmonary embolism	6 (12%)
Recurrent deep vein thrombosis	9 (18%)
Diabetes mellitus	5 (10%)
Arterial hypertension	25 (49%)
Chronic lung disease	5 (10%)
Chronic heart failure	4 (10%)

BMI = body mass index.

**Table 2:** Clinical parameters at admission.

	*N* = 51
Systolic arterial pressure	134 ± 21 mmHg
Diastolic arterial pressure	81 ± 14 mmHg
Heart rate	102 ± 15 per minute
Oxygen saturation without supplementation	93 ± 8%
cTNI	100.0 pg/ml (r. 1.3 to 1291.3)
BNP	462.6 ± 63.9 pg/ml
sPAP by echocardiography	47 ± 3 mmHg + CVP
RV/LV ratio by echocardiography	1.15 ± 0.03
Invasive mean pulmonary pressure	38 ± 11 mmHg
sPESI	1.3 ± 0.7

cTNI = cardiac troponin I; BNP = brain natriuretic peptide; sPAP = systolic pulmonary artery pressure; RV/LV = right ventricular to left ventricular; sPESI = simplified pulmonary embolism severity index.

## Data Availability

The data can be received by request from the authors.

## Supplementary Materials

Supplementary Materials Supplement Main Text 1: ultrasound-assisted catheter-directed thrombolysis, procedure. It describes the short written explanation of the exact ultrasound-assisted catheter-directed thrombolysis procedure using the EkoSonic™ Endovascular System. Venous access was established using a 6 F introducer sheath in the common femoral vein. According to our standard protocol, pulmonary artery pressure and arterial pressure were documented. A digital subtraction angiography (DSA) was performed selectively in the right and left pulmonary artery by contrast injection via a Pigtail catheter to identify thrombus distribution and burden. Thereafter, the catheter was exchanged over a standard 260 cm wire for an EKOSonic™ infusion device and the EkoSonic™ Endovascular System was finally positioned either in one or both pulmonary arteries according to the DSA results. Over the next 15 hours, 11.5 mg of alteplase was continuously administered per catheter, with a rate of 1 mg/h during the first 8 and 0.5 mg/h for the remaining 7 hours. A normal saline coolant was administered at a rate of 35 ml/h via the coolant port of the EKOSonic™ device. Additionally, patients received unfractionated heparin with a target PTT of 69–90 s via the femoral sheath.
