# Supplementary material for: EKOS™ Jena Experience: Safety, Feasibility, and Midterm Outcomes of Percutaneous Ultrasound-Assisted Catheter-Directed Thrombolysis in Patients with Intermediate-High-Risk or High-Risk Pulmonary Embolism
Source: Can Respir J. 2022 Feb 27;2022:7135958. doi: 10.1155/2022/7135958 (PMC8898866; doi:10.1155/2022/7135958)
Supplement: Supplementary Materials — Supplement Main Text 1: ultrasound-assisted catheter-directed thrombolysis, procedure. It describes the short written explanation of the exact ultrasound-assisted catheter-directed thrombolysis procedure using the EkoSonic™ Endovascular System. Venous access was established using a 6 F introducer sheath in the common femoral vein. According to our standard protocol, pulmonary artery pressure and arterial pressure were documented. A digital subtraction angiography (DSA) was performed selectively in the right and left pulmonary artery by contrast injection via a Pigtail catheter to identify thrombus distribution and burden. Thereafter, the catheter was exchanged over a standard 260 cm wire for an EKOSonic™ infusion device and the EkoSonic™ Endovascular System was finally positioned either in one or both pulmonary arteries according to the DSA results. Over the next 15 hours, 11.5 mg of alteplase was continuously administered per catheter, with a rate of 1 mg/h during the first 8 and 0.5 mg/h for the remaining 7 hours. A normal saline coolant was administered at a rate of 35 ml/h via the coolant port of the EKOSonic™ device. Additionally, patients received unfractionated heparin with a target PTT of 69–90 s via the femoral sheath. [file 7135958.f1.docx]

**Supplementary Main Text 1. Ultrasound-assisted catheter-directed thrombolysis, procedure**

Venous access was established using a 6 F introducer sheath in the common femoral vein. According to our standard protocol pulmonary artery pressure and arterial pressure were documented. A digital subtraction angiography (DSA) was performed selectively in the right and left pulmonary artery by contrast injection via a Pigtail catheter to identify thrombus distribution and burden. Thereafter, the catheter was exchanged over a standard 260 cm wire for an EKOSonic^TM^ infusion device and the EkoSonic^TM^ Endovascular System was finally positioned either in one or both pulmonary arteries according to the DSA results. Over the next 15 hours 11,5 mg alteplase were continuously administered per catheter, with a rate of 1 mg/h during the first 8 and 0,5 mg/h for the remaining 7 hours. Normal saline coolant was administered at a rate of 35 ml/h via the coolant port of the EKOSonic^TM^ devce. Additionally, patients received unfractionated heparin with a target PTT of 69-90 s via the
